#  Sofrimento mental e suicídio de trabalhadores e trabalhadoras: compreender para mudar

**DOI:** 10.1590/0102-311XPT186623

**Published:** 2024-01-08

**Authors:** René Mendes

**Affiliations:** 1 Frente Ampla em Defesa da Saúde dos Trabalhadores, Santos, Brasil.; 2 Associação Brasileira de Saúde do Trabalhador e da Trabalhadora, Santos, Brasil.

Tendo assumido o desafio de elaborar uma resenha crítica da obra em epígrafe ^1^, faz-se necessário esclarecer - antes de tudo - de que não o faço apenas como um leitor privilegiado deste livro, mas, também, como testemunha, posto haver participado do evento gerador do livro.

Trata-se, com efeito, da coletânea de textos correspondentes às conferências e palestras do 1º Seminário Internacional Sofrimento Mental e Morte entre Trabalhadores e Trabalhadoras - Transtornos Mentais e Suicídios Relacionados ao Trabalho, realizado em Campinas (São Paulo), em 24 e 25 de maio de 2022. O seminário e o livro resultam do projeto Transtornos Mentais e Suicídios Relacionados ao Trabalho, fruto da parceria entre Ministério Público do Trabalho (PRT 15ª Região) e Universidade Estadual de Campinas (Unicamp).

Espelhando a estrutura do seminário, essa obra coletiva de 273 páginas, 31 autoras e autores, composta por 25 capítulos, distribuídos em seis seções estruturantes, sintetiza dois dias de debates profícuos, ocorridos em ambiente altamente qualificado, de perfil diversificado, participativo e dialógico.

O seminário e o livro resultante do evento mostram, em primeiro lugar, que abordar a complexa problemática do sofrimento e adoecimento mental relacionado ao trabalho, incluindo um significativo destaque para o grave problema dos suicídios, constitui um desafio que requer começar pelo começo: entender que esses graves problemas que afetam a vida e a saúde da classe trabalhadora são socialmente construídos. E essa elucidação e denúncia foram feitas por meio de quatro capítulos reunidos no eixo estruturante *Capitalismo e Destruição do Trabalho: Como o Trabalho Contemporâneo Afeta as Relações Pessoais, Sociais e Coletivas*, que constitui a seção I da obra.

E a destruição do trabalho, que destrói a vida e a saúde de trabalhadoras e trabalhadores, é corretamente abordada como decorrência do modo de produção capitalista, turbinado pela voracidade do capitalismo financeiro mundializado - como explica Ricardo Antunes - e consistente com a ideologia neoliberal e ultraneoliberal, que adquire materialidade discursiva, por exemplo, por meio de modelos gerenciais “paradoxantes” - como bem analisa o professor francês Vincent De Gaulejac em sua fala/texto - cujo alcance destrutivo se alastra amplamente, não poupando a própria estrutura sindical, como reconhece o pesquisador e sindicalista José Reginaldo Inácio. Aliás, trabalhadores e trabalhadoras sindicais vivem sob múltiplas pressões e enfrentam diversos conflitos existenciais, também fruto do próprio projeto neoliberal de destruir o trabalho, destruir trabalhadoras e trabalhadores, e de minar a força dos sindicatos e dos movimentos sociais de resistência. No caso brasileiro, agravados pela aliança do poder executivo, legislativo e judiciário com os interesses do capital. Quem não adoece? Quem sobrevive?

Quatro capítulos subsequentes compõem a seção II, intitulada *Sofrimento Mental no Trabalho Contemporâneo e Perspectivas*.

O eixo estruturante seguinte - *Assédio e Suicídio Relacionado ao Trabalho* - é composto por quatro temas. Destaca-se, pelo seu título dramático, o capítulo 10: *Não se Mate: Aguente Mais um Pouquinho!*, escrito pelo professor Roberto Heloani, uma das referências mais conhecidas sobre o tema do assédio moral e organizacional, ao lado de Margarida Barreto, nome sempre lembrado e reverenciado. Heloani nos ensina que o “*assédio faz adoecer e faz morrer, e por que faz morrer? Estas novas formas de trabalho destroem os coletivos de trabalho, e por isso, já falávamos há um tempo, o ‘assédio’ é produto da solidão. E não é à toa que a tática mais utilizada até hoje em empresas públicas e privadas, grandes e pequenas, multinacionais e nacionais é a do isolamento, que é proposital. Porque quando se isola, se corta a comunicação, o sujeito adoece, e quando ele adoece para a depressão é um pulo*” (p. 112).

Ainda na mesma seção, a professora Luci Praun dedica extenso capítulo à “precarização social e do trabalho” enquanto determinantes de “repercussões contundentes na saúde mental”, e Mauro Salles Machado, bancário e líder sindical, explica o “mecanismo adoecedor” e gerador de transtornos mentais na categoria bancária. “*Precisamos unificar todos os atores que lidam com a saúde dos trabalhadores para construir uma grande frente, na qual esteja evidente que a saúde dos trabalhadores tem lado: os trabalhadores*”, conclama Machado (p. 129). Cleonice Caetano, comerciária e líder sindical, com foco na categoria dos comerciários e de forma emocionada e contundente, utiliza o termo médico “hemolacria” - lágrimas de sangue - para expressar sua dor e indignação, mas também a urgência pela mudança desse quadro inaceitável. Ao terminar sua fala e seu texto, cita uma frase atribuída a Margarida Barreto: “*Combater a violência é uma longa luta, mas aprendemos que a única luta que se perde é aquela que se abandona*” (p. 133).

O problema das subnotificações dos transtornos mentais e suicídio relacionados ao trabalho, no Brasil, ocupa outro dos eixos estruturantes do seminário, e a professora Tânia Araújo trata desse tema com maestria ímpar, no extenso e fartamente ilustrado capítulo 14. Com a autora, aprendemos, também, dois termos fundamentais - aparentes neologismos -, cujos conceitos atravessam a obra inteira, embora apenas explicitados nesse capítulo. São o termo “suicidamento” e o conceito de “mortes por desespero”.

Para Araújo, “*o conceito de suicidamento - eventos que vão encurralando as pessoas em situações das quais elas não conseguem mais criar saídas ou que esgotaram suas capacidades de respostas - pode ser um conceito promissor. Ou seja, concebe-se o suicídio não como um ato da vontade do indivíduo, mas sim como resultante de sucessivas pressões e aviltamento pessoal que levam a essa situação trágica. Com base nesse conceito, admite-se o suicídio a partir de uma rede complexa de determinantes*” (p. 152).

Quatro capítulos compõem a seção V do livro, intitulada *Como os TMSRT (Des)Aparecem nos Serviços de Saúde do Trabalhador*, que pode ser uma pergunta, com respostas sérias ali corretamente denunciadas. Por último, seis capítulos compõem a seção *Painel de Experiências*.

Terminamos esta breve resenha crítica adotando as palavras do professor Roberto Heloani, que expressam o grito de todos os que participaram desse livro: “*Oxalá, no futuro,* [as organizações] *ajam pelo sentido do dever, pelo princípio ético, para que façamos deste país, um país decente, não um país que usa do assédio moral para enlouquecer e adoecer pessoas, e para tirá-las das suas carreiras. Que nós possamos transformar esse país em um país que valha a pena se viver e não tenhamos vergonha do que somos*” (p. 114).

Desse jeito não dá mais! Um grito desesperado. Poderia ser, também, a senha para uma mudança profunda e radical. Essa é a proposta do livro!
